# Occult Hepatitis B Infection among Hemodialysis Patients in Tabriz, Northwest of Iran: Prevalence and Mutations within the S Region

**DOI:** 10.1155/2022/3838857

**Published:** 2022-06-28

**Authors:** Narges Eslami, Vahdat Poortahmasebi, Javid Sadeghi, Reza Ghotaslou, Bahram Niknafs, Hossein Bannazadeh Baghi, Mahin Ahangar Oskouee

**Affiliations:** ^1^Department of Microbiology and Virology, School of Medicine, Tabriz University of Medical Sciences, Tabriz, Iran; ^2^Infectious and Tropical Diseases Research Center, Tabriz University of Medical Sciences, Tabriz, Iran; ^3^Research Center for Clinical Virology, Tehran University of Medical Sciences Tehran, Iran; ^4^Kidney Research Center, Tabriz University of Medical Sciences, Tabriz, Iran

## Abstract

Regardless of the extensive screening for the detection of hepatitis B surface antigen (HBsAg), hemodialysis (HD) patients are still severely at the risk of occult hepatitis B virus infection (OBI), especially in developing countries. OBI is defined as the presence of HBV DNA with undetectable HBsAg in the liver and/or Serum. This study aims to determine the prevalence of OBI in HD patients in East Azerbaijan Province, northwest of Iran, and inquire about the mutations in the detected HBsAg. In this cross-sectional descriptive study, ELISA method assessed serum and plasma samples of 118 HBsAg-negative patients undergoing HD treatment for HBV serological markers (HBsAg and Anti-HBc). Specific primers by nested polymerase chain reaction have been utilized to examine HBV DNA; also, direct sequencing of surface genes was carried out to characterize the viral genotypes and S gene mutations. Finally, followed by real-time PCR, the quantity of viral load in OBI-positive patients was determined. A total of 118 HD patients were included (63.6% were male and 36.4% female), with an overall mean age of 60.8 ± 12.8 years old. The prevalence of antihepatitis B core antibody (Anti-HBc) in the study population was 26.3% (31/118). Five patients (4.2%) were positive for HBV DNA and labeled OBI-positive; their plasma HBV-DNA load was less than 100 IU/ml. Following the phylogenetic analysis, the samples with OBI roughly belonged to genotype D, subtype ayw2 and only two had mutations within the S 'gene's major hydrophilic region (MHR), including T123I, C124F, and P127T. This study reports the prevalence of OBI in the HBsAg-negative HD patients being at a rate of 4.2%, which can be a clinically vital consideration in this region. HBV serologic screening approaches need to be renewed to cover nucleic acid testing in the setting of hemodialysis and all the other high-risk groups associated with it (i.e., blood and organ donors).

## 1. Introduction

Hepatitis B virus (HBV) is regarded as a severe public health issue in developed and developing countries. Approximately 257 million chronic HBV infection cases around the globe, by and large, are at risk of liver disease [1]. Even though an effective vaccine to prevent HBV infection is currently at hand, HBV-related acute and chronic liver disease remain one of the prime causes of mortality [2]. More than 780, 000 people annually die due to HBV infection-related complications [3]. Since the 1980s, it has been known that HBV may be spreading and transmitting via blood products and organs donated by individuals who are negative for HBsAg [4]. This phenomenon later became an occult hepatitis B virus infection (OBI) [5]. OBI refers to the presence of replication-competent HBV DNA, including the presence of episomal HBV covalently closed circular DNA (cccDNA) in the liver and/or at low levels of serum (not greater than 10^4^ copies/mL) in individuals who were reported negative for hepatitis B surface antigen (HBsAg) utilizing currently available assays [6]. Patients with OBI are classified into seropositive and seronegative groups based on the presence or absence of antibodies against the hepatitis B virus (anti-HBc and anti-HBs) [5, 6].

In developing countries, particularly in the Middle East, end-stage renal disease (ESRD) is a significant concern [7]. Hemodialysis (HD) units and chronic HD patients are mainly predisposed to blood-borne viral infections, especially HBV infections caused by frequent blood transfusions, shared dialysis equipment, and reduced host immunity following kidney transplantation [8]. To avoid such infections, hemodialysis centers employ several methods such as vaccination of patients and staff, segregation of HBsAg-positive patients with their equipment from HBV-sensitive patients, and regular infection control strategies [9]. The clinical importance of OBI remains to be inadequately clarified. However, from the perspective of public health in patients going through HD, the risk of HBV transmission and the probability of viral reactivation following OBI due to their immunosuppressive conditions are considered serious concerns [10]. The most severe complication of OBI is the aggravation of chronic liver disease and fibrosis, which extensively raises the risk of hepatocellular carcinoma by eight times [11]. In Iran, since 1975, all hemodialysis patients have had a biannual serologic examination of serum HBsAg, anti-HBs, and hepatitis C virus antibody (anti-HCV) performed by ELISA techniques to the guidelines of the Ministry of Health (MOH) [12]. Due to chronic renal failure, HD patients suffer from a weakened immune system, minimal inflammation, and mild clinical symptoms, leading to abnormal serological development and decreased vaccine response [13].

Since the specific mechanism of OBI is vague, several scientific theories have been presented. One of the well-known causes of OBI is mutations in the “a” determinant of HBsAg [14]. The S domain encoded by the S open reading frame of the HBV genome is the main component for HBsAg production. The important antigenic loop located in HBsAg is “a” determinant region (amino-acids 124–147). This region significantly induces protective immune cells as the main epitope [15,16]. Variations and mutations within the “a” determinant include P120S/E, K122R, T126A, P127T, Q129H/R, L134S, K141E, P143S, D144A/E/V, and G145R/A, which have been mainly linked to the change of antigenicity and reduced HBsAg production [17]. These substitutions, in essence, can vigorously affect the process of HBsAg screening through the serological assays (diagnostic-escape) and the possibility of vaccine escape [17,18]. Iran has moderate and low endemicity, with a 2.2% prevalence of HBV infection in the overall population [19,20]. The prevalence of OBI among HD patients in the world has ranged from 0% to 58%, while in Iran, it has fluctuated between 0% and 11% (2.49%) [21,22]. This divergence in distributions was mainly dependent on the endemicity of HBV in each region, specimen type, sample size, and methods used for the studies. Unfortunately, East Azerbaijan's most important medical center in northwestern Iran offers relatively limited information on OBI screening in HD patients. In the present study, by utilizing serological and molecular techniques, the incidence of OBI was fully examined in a group of HD patients, as well as the effects of possible genomic mutations associated with gaining OBI for the first time in this region.

## 2. Materials and Methods

### 2.1. Study Population and Samples Gathering

This cross-sectional investigation comprised 118 blood samples of HD patients referred to the Imam Reza Hemodialysis Unit, the largest hemodialysis center in Northwest Iran, between October 2020 and January 2021. The inclusion criteria included patients who had been on three weekly hemodialysis sessions for at least 12 months continuously, and the exclusion criteria were positive for HBsAg. First, informed consent and permission were obtained from all patients. Then 10 ml whole blood samples were collected (before the initiation of hemodialysis). Data such as age, gender, sociodemographic, laboratory reports, and significant clinical status were gathered from the documented records using a standardized questionnaire. The obtained samples were transported to the virology laboratory of Tabriz University of Medical Sciences, aliquoted and stored at −70°C until tested. The Ethical Committee utterly approved the study protocol and this human work standard of the Tabriz University of Medical Sciences.

### 2.2. Ethical Consideration

The Ethical Committee of Tabriz University of Medical Sciences, with the protocol number: IR.TBZMED.REC.1399.981 reviewed and authorized the study. All hemodialysis patients offered their informed consent before blood collection. The methods were carried out in agreement with the principles and propositions established in the Declaration of Helsinki for all human or animal subjects.

### 2.3. Biochemical and Serological Tests

Venous blood samples of all participants were separated, and the serum levels of alanine aminotransferase (ALT), aspartate aminotransferase (AST), and alkaline phosphatase (ALP) were precisely measured by an automated analyzer (BT 3000) within 48 hours of blood gathering. In addition, the HBV-related serological markers, consisting of HBsAg and anti-HBc, were measured utilizing an ELISA (Dia. Pro, Italy). Finally, consistent with the manufacturing procedure, all samples were reevaluated with another ELISA kit (Acon, USA).

### 2.4. HBV-DNA Extraction

HBV DNA was extracted from 200 *μ*l patients' plasma using the Viral Nucleic Acid Extraction Kit (GBC viral DNA/RNA Extraction Kit, Taiwan) according to the manufacturer's recommendation along with known positive and negative controls in each step. In the final step, DNA was eluted using 50 *μ*l of elution buffer. The extracted DNA was kept at −70°C until it was used.

### 2.5. Nested Polymerase Chain Reaction (PCR)

**Figure 1:**
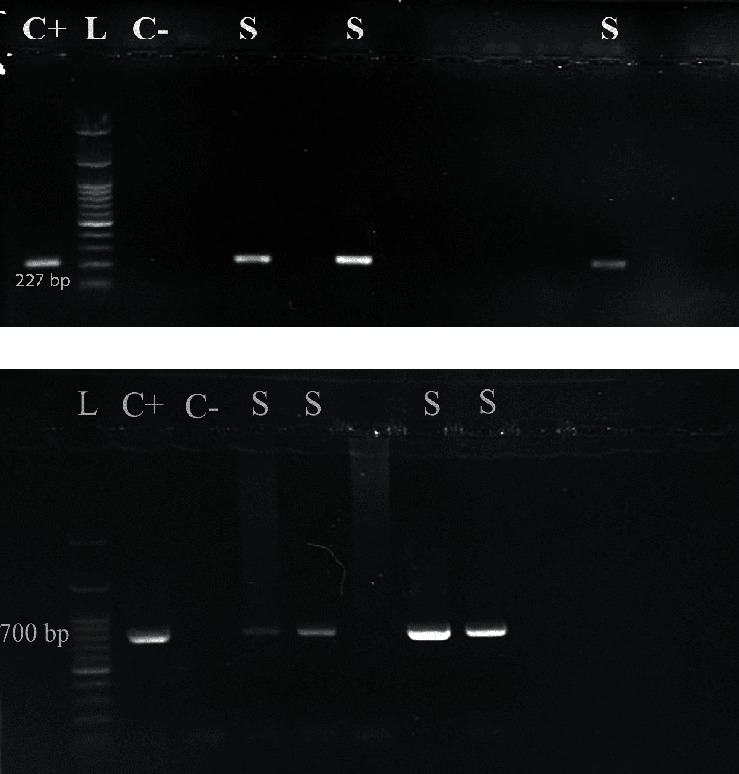
(a) Agarose gel electrophoresis of the nested PCR for OBI. C+: positive control; L: 100 bp DNA ladder; C−: negative control; S: OBI-positive samples (227 bp). (b) Agarose gel electrophoresis of the second round of nested PCR for HBsAg amplicon with a length of 700 bp. C+: positive control; L: 100 bp DNA ladder; C−: negative control; S: amplified fragment for sequencing (700 bp).

Aside from serological tests, HBV DNA was checked out in all samples through sensitive nested PCR using primers recommended by the Taormina OBI International Committee meeting to determine OBI; for instance, for the amplification of the highly conserved S gene, a 227 bp fragment of its partial sequence was used (detailed information can be accessed in Table 1) [5]. Subsequently, the full-length sequence (681 bp) of the HBsAg gene for all the positive samples was amplified using specific primers to determine mutations, genotypes, and subtypes (Table 2) [23]. The two rounds of nested PCR were deliberately administered to amplify the HBsAg region of HBV DNA. The thermal-cycling profile consisted of Taq activation at 95°C for 5 min, followed straightly by 35 cycles of PCR amplification utilizing the temperatures as below denaturation at 94°C for 30 s, annealing at 60°C for 45 s, and extension at 72°C for 45 s, with a final extension 72°C for 10 min. Cycling conditions for the second round of PCR were similar to the first round amplification profile; however initial denaturation was 95°C for 15 min and annealing at 58°C for 45 s. The products of the second PCR round were examined by electrophoresis and run on a 1% agarose gel (Figures 1(a) and 1(b)).

### 2.6. Quantitative Real-Time PCR (qRT-PCR) for HBV DNA

A qRT-PCR was carried out by utilizing GeneProof PCR Kit (Brno, Czech Republic) for HBV viral load quantification (IU/mL) of all positive standard PCR samples in line with the manufacturers' instructions. The detection limit (LoD) of the assay maintained by the manufacturer was determined to be 36.9792 copies/mL.

### 2.7. Sequencing of HBsAg

The full-length HBsAg amplified regions of HBV DNA derived from the second round of nested PCR (681 bp) were sequenced bilaterally through the ABI-3130 Genetic Analyzer machine (DNA Sequencer, Applied Biosystems 3130, Foster City, CA, USA). The HBsAg sequences were transferred to the Chromas software (version 2.1.1.). They were thoroughly compared and edited, and then extra sequences were wholly taken out. Finally, all full-length HBsAg sequences (681 bp) alignments were conducted via BioEdit software (version 7.0.5.3.). Thus, to evaluate amino acid/nucleotide substitution in the HBsAg region, the strain sequence (accession number GQ183486) obtained from the GenBank site was considered the reference and the sequences were compared with it. HBsAg sequencing was used to determine the HBV genotypes and subtypes. Genotyping was done using HBV genotypes A to H reference sequences and sequence analysis of full-length surface genes related to OBI samples. Amino acid replacement from K to R at sequences 122 and 160, respectively, produces the divergence between subtype-specific determinants d/y and w/r [24,25]. All five sequences have been set forth to the GenBank under accession numbers OK106296-OK106300.

### 2.8. Statistical Analysis

We have taken advantage of SPSS Statistics version 22 software (SPSS Inc, Chicago, Illinois, USA) to analyze the whole data. Descriptive statistics (Numerical variables) are expressed as the mean ± standard deviation (SD). In addition, the frequencies were indicated as an absolute number and a percentage.

## 3. Results

### 3.1. Demographic and Clinical Characterization

The present study collected 118 whole blood samples from HD patients. All of the samples were evaluated for serological and molecular analysis. The demographic, clinical, and virological characteristics of the study population are summarized in Table 3. The studied patients were aged 26 to 87 years old (60.8 ± 12.8 years). Males constituted 75/118 (63.6%), and females were 36.4%. The mean month of hemodialysis duration for all patients was 28.7 ± 15.8, and 53.4% (63/118) had gained not less than one transfusion of packed red blood cells. The HBV infection-related serological markers revealed that no HBsAg-positive cases were found. In total, 31 individuals 26.3% were anti-HBc-positive, which indicated previous infection with HBV. However, due to a discernible band in the nested run of a partial segment of the S region, HBV DNA was found in 5/118 (4.2%) of the patients considered positive for OBI (Figure 1(a)). Four OBI-positive patients were positive for anti-HBc, and one without serological markers had a history of a kidney transplant. The HBV DNA levels of all 5 OBI-positive samples were <10^4^ copies/ml (Table 4). The mean age of the OBI patients was 65 ± 10.8 years, comprising four males and one female. In all patients in addition to OBI patients, mean ALT liver enzyme values were 15.9 ± 7.6 IU/L and 13.4 ± 7.7 IU/L, respectively. The mean AST level was 25.3 ± 11.2 IU/L and 23.8 ± 9.9 IU/L in total and OBI patients. These results indicate that both liver function markers were in the normal range in HD patients who tested positive or negative for OBI. The levels of ALP in studied patients and OBI-positives were 311.7 ± 205.7 IU/L and 341.2 ± 158 IU/L, respectively. However, they were pretty high, which indicated more damage to the bile ducts than the liver.

### 3.2. Molecular Features and Sequencing Analysis of HBsAg

**Figure 2:**
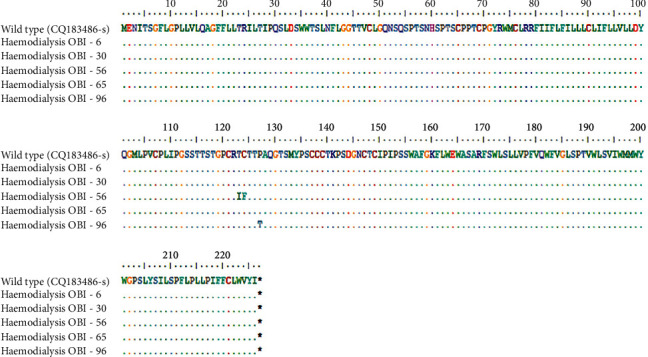
Representation of five HBsAg sequences (681 nucleotides) acquired from OBI-positive samples. The amino acid substitutions are reported in different colors by the BioEdit program. The top sequence belongs to GQ183486 HBsAg from an Indian genotype D isolate.

**Figure 3:**
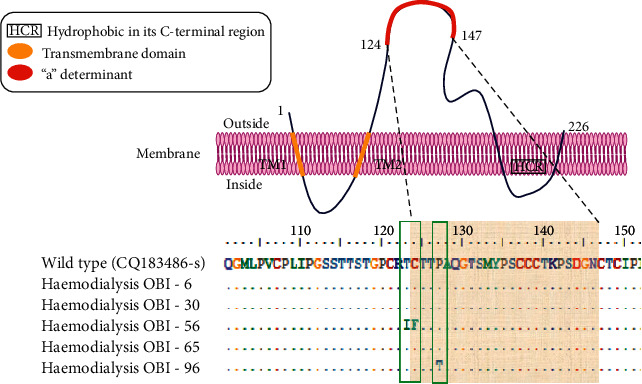
The amino acid sequence alignment of the HBV S gene as compared to the reference sequence. The OBI number is shown next to each patient sample separately. A pink box indicates the sequence of “a” determinant. Dots represent the identical amino acids as in the reference sequence, whereas letters represent the variations.

Based on the direct sequencing of the full-length surface gene with the 681 nucleotides encoding HBsAg and the alignment of the amino acid sequences (226 aa) for PCR positive-OBI cases (Figures 1(b) and 2), solely two OBI patients, OBI No. 56 and 96, had mutations within the MHR and the “a” determinant (amino acid position 120–150 and 124–147, respectively) (Figure 3). No mutations were found in three other OBI participants (3/5), which does not reject the possibility of transmission between them. However, contamination inside the laboratory cannot be ruled out. OBI 56 was discovered to have mutations of Thr123Ile (T123I) and Cys124Phe (C124F), while the other had a mutation of Pro127Thr (P127T) in the “a” determinant of HBsAg (Figure 3, Table 4). These substitutions, particularly in the “a” determinant of HBsAg, could result in decreased HBsAg antigenicity. Accordingly, these changes might be to blame for HBsAg detection evasion. All HBV strains belong to HBV genotype D. According to the direct sequencing of the full-length S region of HBV DNA, all five OBI samples embodied arginine (R) in position 122 and lysine (K) in position 160, indicating the HBV subtype ayw2.

## 4. Discussion

HBV is highly transmittable, roughly a hundred times more contagious than HIV. Even in its occult form, it can survive for up to 7 days on surrounding surfaces [26,27]. In developing countries, the rate of HBV infection in hemodialysis patients ranges from 11% to 15% [28]. In 2012, an estimated 2.1 million individuals underwent HD treatment worldwide, with a forecast two-fold rise (5.4 million) by 2030 [29]. Although the transmission of OBI in blood products of donors is reported to be low [30], a minimum level of viremia for transmissibility of OBI in the hemodialysis setting needs to be surveyed in further studies. Studies have shown that the lack of HBV-specific T cell growth in OBI patients might be inadequate for developing protective memory cells, probably due to a low HBV viral load [31]. The viral load in our OBI cases was less than 10^4^ copies/mL, confirming the OBI definition [6].

Iran is classified as having low-intermediate HBV prevalence with a rate of 2.2% from 1990 to 2016 [19,20]. The prevalence of 4.2% OBI in this cross-sectional study is in line with the endemicity of HBV in the region's general population. It is estimated that about 20% of OBI patients are regarded to be negative for HBV antigens and antibodies since the onset of infection or may have been lost gradually. In comparison, 80% are positive for one or more anti-HBV antibodies [6]. Anti-HBc, a nonprotective antibody that cannot neutralize or inhibit HBV, was employed as a marker for previous exposure or chronic HBV infection [32]. It might be found in severe, chronic, and resolved HBV infections in almost all patients for their entire lives [32]. It can also be identified in OBI-positive individuals with a low HBV DNA viral load [33]. In the case of OBI, the seronegative status might be the source of infection transmission. Despite invasive sampling, the detection of HBV DNA in the liver biopsy is the gold standard technique for diagnosing seronegative OBI [6]. But it has numerous disadvantages, including sampling inaccuracy, expensiveness, and the risk of consequences. Anti-HBc prevalence was 26.3% in this study, and four of the OBI cases were seropositive in this marker. This matter is in line with the results of Mandour et al., who found that OBI is more frequent among seropositive individuals [34].

Over the past decade, various studies have been conducted in different parts of Iran and the world to investigate the prevalence of OBI in the hemodialysis population. According to the sample size or type, diagnostic methods, and endemicity of the HBV, their results were quite different in each region. Kalantari et al. have found the frequency of positive anti-HBc in hemodialysis patients in Isfahan, a central city of Iran, at 32/400 (8%) and all of them were found to be negative for HBsAg and the presence of HBV DNA (0%) [35]. In Samiei et al. study, HBV DNA was identified by nested PCR in 4/84 (4.7%) hemodialysis patients in Ahvaz, located in southern Iran, and all the OBI cases were seropositive [36]. The prevalence of OBI in Tehran, the capital of Iran, was described as 0.5% (1/200). Ranjbar et al. expressed that the very low prevalence of HBV DNA may be related to the accuracy of regular and timely vaccination in the hemodialysis groups [37]. Eltom et al. during a systematic analysis which was conducted on a 10-year study in Sudan, stated that the pooled prevalence of OBI in the hemodialysis group was 13.4%, which was relatively high due to its endemicity [38]. Egypt with a (2–8%) prevalence of HBsAg is considered as an intermediated endemic region, while Helaly et al. have recognized 32% of hemodialysis study groups as OBI-positive and all patients have anti-HBc positively [39]. Fontenele et al. declared the presence of HBV DNA in Brazilian hemodialysis patients was 2.3% (7/301) which could be primarily due to interference with the simultaneous presence of HCV [40].

A previous study claimed a high incidence of occult HBV infection (31 of 195, 16%) among subjects with normal ALT levels in South Korea [41]. Likewise, the results of this study illustrate a similar situation. All of the HD patients in our research, including those with OBI, exhibited normal liver enzyme activity. The presence of uremic factors is primarily affected by blood urea nitrogen (BUN) or creatinine. These blood markers are a disruptive problem in all HD patients. It has been proposed that this could inhibit these enzymes' activity, resulting in a reduction of aminotransferases in HD patients [42]. The other explanation is about the immunological status of HBV infection or, in other words, the actions of specific CD4 and CD8 lymphocytes which could lead to an increase in AST and ALT levels. In contrast, in HD patients with HBV infection, the actions of specific viral immune system lymphocytes, including CD4 and CD8, remain inadequate and decline; thus, the level of AST and ALT will not be elevated [43]. According to the observations of Mohraz et al. ALT elevation is potently linked to the existence of anti-HCV antibodies but was not correlated with occult HBV infection [44].

In a meta-analysis study in 2016, the prevalence of HBV in men was revealed to be more than 1.3 times that of females [45]. However, in the present study, four out of five OBI-positive cases were also male. Although the number of male HD patients in this study was more than females, the observation found that females were more likely than men to create antibodies against hepatitis B in response to infection. At the same time, males were more susceptible to developing chronic hepatitis B infection [46].

One of our seronegative OBI patients got a kidney transplant. Research by Mexicans also found that two out of 73 kidney recipients tested positive for HBV DNA after a three-month follow-up using high-sensitivity molecular methods [47]. Because of various epidemiological encounters, such as HD, transfusions, or even transplantation operations, renal transplant patients might become infected with HBV. At the beginning of 1996, all blood donors in Iran were tested for hepatitis infection and the immunization program. Despite this, blood donations continue to be one of the most essential sources of hepatitis virus infection transmission, and the majority of our OBI patients received at least one transfusion of red blood pack. In addition, some OBI carriers may not be detected by simple routine ELISA screening in blood donors, increasing the chance of virus transition through blood transfusions.

According to our observation, HBV in study participants belongs to genotype D, predominantly subgenotype D2 and subtype ayw2 (results not shown). This is in line with the preceding study that reported genotype D as the most familiar HBV genotype in Iran [48]. The HBsAg test is the most common way to determine the presence of HBV infection. However, HBsAg variants emerge as an escape strategy during infection. Antibodies can be recognized and neutralized by the epitopes of MHR, which contain the leading binding site for antibodies (anti‐HBs) against HBsAg for lifelong immunity. The amino acid alignment of sequences from OBI patients in this study shows differences across the MHR and “a” determinant region of the S gene, spanning amino acids 120 to 150 with the reference sequence of genotype D. The incidence of all the variations T123I, C124F, and P127T in the MHR of two OBI participants has been observed previously [44,49]. Single amino acid substitutions in the “a” determinants have previously been found to misdiagnose by HBsAg tests (diagnostic-escape) and/or avoidance of detection by the host's immune system (vaccine escape) [49]. Due to the role of proline as an amino acid, which is needed to facilitate protein folding and loop integration [50], local deformation caused by P127T substitution may potentially affect the integrity of HBsAg antigenic loops. which has a fundamental role in HBsAg-antibody interaction. The conserved residue of T123 is required to maintain antigenic loops and recognize HBsAg by monoclonal antibodies [44]. Nonetheless, T123I can disrupt the integrity of HBsAg antigenic loops by preventing the formation of a disulfide bond of C124, which is located close to it. As a result of these conformational changes, HBsAg levels in OBI-positive individuals may be reduced by trapping it in the endoplasmic reticulum (ER) and HBsAg collapsing in the budding process. However, our finding did not detect the mutation of glycine to arginine located in the 145th codon of HBsAg (sG145R), which has been commonly correlated to OBI and vaccine scape globally [49]. This polymorphism antigenicity of the HBV surface gene, especially in MHR and the “a” determinant region, may affect recognition and change the expression of epitopes. So, the diagnosis of HBV infection may be missed if only HBsAg is measured [51].

The study's limitations include the coincidence of the SARS-CoV-2 pandemic with sample gathering and the lack of clinical information on COVID-19's history and plasma therapy. According to research, plasma, rather than red blood cells, appears to have a more efficient role in transmitting HBV [52]. After a few years, follow-up of OBI patients suggested investigating the status of viral genome integration, especially in those without mutation, which can clarify the other molecular characterization of OBI. Given the prevalence of hepatitis C in the research location, the possibility of coinfection and its influence on immune system dysfunction cannot be denied and should be considered in further studies.

## 5. Conclusion

We have indicated the prevalence, genotype, and sequence analysis of occult HBV infection in hemodialysis patients in the capital city of East Azerbaijan, northwest of Iran. Hemodialysis patients are at serious risk of HBV transmission and reactivation due to using shared equipment and immunosuppressants after kidney transplantation. Therefore, precise identification of HBV is crucial for infection management and treatment. Based on our findings and prior research, OBI should be considered a potential cause of HBV infection in HD patients. To detect likely OBI, serological indicators of HBV should be confirmed by molecular assays such as PCR and real-time PCR, especially among anti-HBc positive hemodialysis patients. Hence, a screening system based on molecular techniques could be a recommended course of action. This might include the safety of blood products used in transfusions and kidney transplant candidates, which are connected to viral transmission in hemodialysis.

## Figures and Tables

**Table 1:** Primer sets for the detection of OBI through nested PCR.

HBV S region	Nucleotide position (HBV)	Fragment size (bp)
Ex: S1-F: 5′-CATCAGGATTCCTAGGACCCCT-3′	[168–189]	**290**
Ex: S4-R: 5′-AGGACAAACGGGCAACATAC-3′	[478–458]	
In: S2-F: 5′-CTTGTTGACAAGAATCCTCACA-3′	[214–235]	**227**
In: S3-R: 5′-CCAACAAGAAGATGAGGCATA-3′	[442–420]	

Ex, external sequence; In, internal sequence; HBV, hepatitis B virus; bp, base pair; F, forward strand; R, reverse strand.

**Table 2:** Sequences and sizes of primers used for full-length hepatitis B surface protein.

Primers for HBV S-gene region	Nucleotide position (HBV)	Fragment size (bp)
*First round*		
S1-F: 5′-CCTGCTGGTGGCTCCAGTTC-3′	[56–75]	**1000**
S2-R: 5′-CCACAATTCKTTGACATACTTTCCA-3′	[979–1003]	

*Second round*		
S6-F: 5′-GCACACGGAATTCCGAGGACTGGGGACCCTG-3′	[133–146]	**700**
S7-R: 5′-GACACCAAGCTTGGTTAGGGTTTAAATGTATACC-3′	[823–857]	

HBV, hepatitis B virus; bp, base pair; F, forward strand; R, reverse strand.

**Table 3:** Clinical features of study population.

Characteristics	Total	HBV DNA + (OBI)
	(*n* = 118)	(*n* = 5) 4.2%
Sex, male/female (%)	75/43 (63.6)	4/1 (80)
Age (years, mean ± SD)	60.8 ± 12.8	65 ± 10.8
Duration of HD (months, mean ± SD)	28.7 ± 15.8	42 ± 17.2
History of transfusion (*n*, %)	63, 53.4	4, 80
ALT (IU/L)	15.9 ± 7.6	13.4 ± 7.7
AST (IU/L)	25.3 ± 11.2	23.8 ± 9.9
ALP (IU/L)	311.7 ± 205.7	341.2 ± 158
Consumption of alcohol/smoking (%)	0.8/21.2	0/20
Hepatitis B vaccination (*n*, %)	95, 80.5	4, 80
Anti-HBc ab+ (*n*, %)	31, 26.3	4, 80
History of member transplant (*n*, %)	8, 6.8	1, 20
History of diabetes (*n*, %)	59, 50	2, 40
History of high blood pressure (*n*, %)	84, 71.2	3, 60
Booster dose of vaccine (*n*, %)	13, 11	0

SD, standard deviation; *n*, number; HBV DNA, hepatitis B virus deoxyribonucleic acid; HD, hemodialysis; IU/L, international unit per liter; ALT, alanine amino transferase; AST, aspartate amino transferase; ALP, alkaline phosphatase; anti-HBc, hepatitis B core antibody.

**Table 4:** Molecular characteristics of OBI samples.

Sample code	Sex	Age (years)	HBV DNA (copies/mL)	Anti-HBc	Surface mutations
OBI 6	Male	58	1050	+	—
OBI 30	Male	69	3730	−	—
OBI 56	Female	50	4670	+	T123I, C124F
OBI 65	Male	77	960	+	—
OBI 96	Male	71	2900	+	P127T

## Data Availability

The data that support the findings of this study are available and included within the article.
